# Evaluation of the impact of cardiac implantable electronic devices on cine MRI for real‐time adaptive cardiac radioablation on a 1.5 T MR‐linac

**DOI:** 10.1002/mp.17438

**Published:** 2024-10-04

**Authors:** Osman Akdag, Stefano Mandija, Pim T. S. Borman, Paris Tzitzimpasis, Astrid L. H. M. W. van Lier, Rick Keesman, Bas W. Raaymakers, Martin F. Fast

**Affiliations:** ^1^ Department of Radiotherapy University Medical Center Utrecht Utrecht The Netherlands; ^2^ Computational Imaging Group for MR Diagnostics and Therapy Center for Image Sciences University Medical Center Utrecht Utrecht The Netherlands; ^3^ Department of Radiotherapy Radboud University Medical Center Nijmegen The Netherlands

**Keywords:** cardiac radiotherapy, MRI‐guided radiotherapy, real‐time

## Abstract

**Background:**

Stereotactic arrhythmia radioablation (STAR) is a novel treatment approach for refractory ventricular tachycardia (VT). The risk of treatment‐induced toxicity and geographic miss can be reduced with online MRI‐guidance on an MR‐linac. However, most VT patients carry cardiac implantable electronic devices (CIED), which compromise MR images.

**Purpose:**

Robust MR‐linac imaging sequences are required for cardiac visualization and accurate motion monitoring in presence of a CIED during MRI‐guided STAR. We optimized two clinically available cine sequences for cardiorespiratory motion estimation in presence of a CIED on a 1.5 T MR‐linac. The image quality, motion estimation accuracy, and geometric fidelity using these cine sequences were evaluated.

**Methods:**

Clinically available 2D balanced steady‐state free precession (bSSFP, voxel size = 3.0 × 3.0 × 10 mm^3^, T_scan_ = 96 ms, bandwidth (BW) = 1884 Hz/px) and $T_{1}$‐spoiled gradient echo ($T_{1}$‐GRE, voxel size = 4.0 × 4.0 × 10 mm^3^, T_scan_ = 97 ms, BW = 500 Hz/px) sequences were adjusted for real‐time cardiac visualization and cardiorespiratory motion estimation on a 1.5 T Unity MR‐linac (Elekta AB, Stockholm, Sweden), while complying with safety guidelines for MRI in presence of CIEDs (specific absorption rate < 2 W/kg and $\frac{dB}{dt}<$ 80 mT/s). Cine acquisitions were performed in five healthy volunteers, with and without an implantable cardioverter– defibrillator (ICD) placed on the clavicle, and a VT patient. Generalized divergence‐curl (GDC) deformable image registration (DIR) was used for automated landmark motion estimation in the left ventricle (LV). Gaussian processes (GP), a machine‐learning technique, was trained using GDC landmarks and deployed for real‐time cardiorespiratory motion prediction. $B_{0}$‐mapping was performed to assess geometric image fidelity in the presence of CIEDs.

**Results:**

CIEDs introduced banding artifacts partially obscuring cardiac structures in bSSFP acquisitions. In contrast, the $T_{1}$‐GRE was more robust to CIED‐induced artifacts at the expense of a lower signal‐to‐noise ratio. In presence of an ICD, image‐based cardiorespiratory motion estimation was possible for 85% (100%) of the volunteers using the bSSFP ($T_{1}$‐GRE) sequence. The in‐plane 2D root‐mean‐squared deviation (RMSD) range between GDC‐derived landmarks and manual annotations using the bSSFP (T_1_‐GRE) sequence was 3.1–3.3 (3.3–4.1) mm without ICD and 4.6–4.6 (3.2–3.3) mm with ICD. Without ICD, the RMSD between the GP‐predictions and GDC‐derived landmarks ranged between 0.9 and 2.2 mm (1.3–3.0 mm) for the bSSFP (T_1_‐GRE) sequence. With ICD, the RMSD between the GP‐predictions and GDC‐derived landmarks ranged between 1.3 and 2.2 mm (1.2–3.2 mm) using the bSSFP (T_1_‐GRE) sequence resulting in an RMSD‐increase of 42%–143% (bSSFP) and −61%–142% (T_1_‐GRE). Lead‐induced spatial distortions ranged between −0.2 and 0.2 mm (−0.7–1.2 mm) using the bSSFP ($T_{1}$‐GRE) sequence. The 98^th^ percentile range of the spatial distortions in the gross target volume of the patient was between 0.0 and 0.4 mm (0.0–1.8 mm) when using bSSFP ($T_{1}$‐GRE).

**Conclusions:**

Tailored bSSFP and $T_{1}$‐GRE sequences can facilitate real‐time cardiorespiratory estimation using GP trained with GDC‐derived landmarks in the majority of landmark locations in the LV despite the presence of CIEDs. The need for high temporal resolution noticeably reduced achievable spatial resolution of the cine MRIs. However, the effect of the CIED‐induced artifacts is device, patient and sequence dependent and requires specific assessment per case.

## 1 INTRODUCTION

Stereotactic arrhythmia radioablation (STAR), also referred to as cardiac radioablation, is a novel noninvasive treatment strategy for treating patients with refractory ventricular tachycardia (VT). During STAR a single high‐dose fraction (typically 1 × 25 Gy) is delivered to the treatment target (i.e., the VT isthmus) for VT termination.^1^, ^2^, ^3^ STAR is a salvage treatment approach for VT patients who are not eligible for catheter ablation.^4^, ^5^

An increasing number of VT patients are treated using STAR on conventional C‐arm and/or robotic linacs with encouraging early results,^6^, ^7^ but accurate treatment target visualization and motion mitigation remains challenging and can lead to large treatment volumes.^8^ Cardiac (sub‐)structures and adjacent mediastinal organs‐at‐risk (OARs) are highly dose sensitive and it is therefore beneficial to keep the treatment volume at a minimum.^9^, ^10^ Computed tomography is mainly used for imaging and radiotherapy treatment planning, but accurate visualization of the treatment target is challenging due to (soft‐tissue) contrast limitations of x‐ray‐based imaging. Additionally, the treatment target is subjected to cardiorespiratory motion (unable to be captured using x‐ray‐based imaging approaches), which induces dose uncertainties and therefore amplifies concerns about geographic miss and toxicity.^11^

The aforementioned challenges can potentially be addressed by performing the STAR treatment procedure with MRI‐guidance. The treatment target can be accurately visualized with either a diagnostic MRI scanner and/or a hybrid MRI and linear accelerator (MR‐linac) system due to the superior soft‐tissue image contrast. Online MRI‐guidance on an MR‐linac can be used to cope with intra‐fraction cardiorespiratory motion of the treatment target, which we demonstrated experimentally in previous studies.^12^, ^13^

VT patients are likely to carry a cardiac implanted electronic device (CIED), such as a pacemaker or implantable cardioverter– defibrillator (ICD), which introduces safety and imaging challenges for an MRI‐guided STAR procedure. The presence of a CIED used to pose a safety challenge for MRI examinations and patients with a CIED were historically excluded from MRI examinations. However, in today's practice, safety guidelines have evolved to include patients with CIEDs in MRI examinations, which are also increasingly labelled as MRI‐conditional.^14^, ^15^

Additionally, the homogeneity of the static magnetic field ($B_{0}$) is locally compromised by the presence of a CIED. Consequently, the acquired MRI images become impaired by severe artifacts (i.e., structure visualization impairments and inhomogeneous signal patterns) and introduce spatial distortions.^16^, ^17^ Therefore, the accurate visualization of a treatment target can be compromised during online MRI‐guided radiotherapy in presence of a CIED, which raises concerns regarding geographical miss during online motion‐mitigated dose delivery.

The impact of the presence of metallic implants during MR‐guided radiotherapy has been investigated by Keesman et al. and Van Lier et al. using the 1.5 T Unity MR‐linac (Elekta AB, Stockholm, Sweden). The geometric fidelity was investigated in prostate cancer patients using static field mapping to test the feasibility of MRI‐guided radiotherapy and tailor clinical workflows for prostate cancer patients with unilateral hip implants.^18^, ^19^ More recently, multi‐institutional experiences were reported in which the impact of the presence of CIEDs on MR‐guided radiotherapy were investigated in pancreatic and prostate cancer patients,^20^, ^21^ which showed that it was feasible to treat these patients with MR‐guided radiotherapy on a 1.5 T MR‐linac. Supporting evidence for the treatment of patients carrying a CIED with thoracic lesions using MR‐guidance at 1.5 T is, however, marginal.

Cardiac treatment targets are increasingly being treated on MR‐linac systems, but two reported cases included patients carrying a CIED.^22^, ^23^ Gach et al. and Mayinger et al. locally developed safety protocols for MRI‐guided radiotherapy in cardiac patients with only respiratory motion mitigation using the 0.35 T MRIdian MR‐linac (ViewRay, Oakwood Village, Ohio, USA) for the treatment of cardiac fibroma and VT, respectively. Mayinger et al. have also investigated the geometric fidelity in the treatment target region by creating an experimental simulation in presence of a CIED.

To achieve a highly conformal dose delivery during MRI‐guided STAR for VT patients with a CIED, high frequency (preferably >10 Hz) cine imaging sequences are required to accurately resolve and mitigate cardiorespiratory motion in real‐time on the MR‐linac. It is also required to investigate the geometric image fidelity in the thorax due to the local CIED‐induced disruption of the static magnetic field, which has the potential to negatively impact on accurate treatment planning and adaptation processes.

To this end, we adapted two clinically available cine MRI sequences for real‐time cardiorespiratory motion monitoring on the 1.5 T Unity MR‐linac in presence of CIEDs. The image quality and performance of these cine sequences for real‐time motion estimation were assessed in a phantom and in vivo (healthy volunteers and VT patient). The geometric fidelity of the cine MRI sequences in presence of CIEDs was also investigated by including field mapping during the imaging experiments.

## 2 METHODS

### 2.1 Cine MRI sequence acquisition

Two distinct single‐slice 2D cine MRI sequences were tailored for cardiorespiratory motion estimation on the 1.5 T Elekta Unity MR‐linac in free‐breathing (i.e., no motion compensation during image acquisitions). The performance of the cine MRI sequences was assessed for real‐time adaptive radiotherapy purposes in a phantom (Section 2.1.1), healthy volunteers (Section 2.1.2) and a VT patient (Section 2.1.3). First, a balanced steady‐state free precession (bSSFP) 2D cine sequence (TR/TE = 2.8/1.4 ms, flip angle (FA) = 50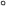, acquisition voxel size = 3.0 × 3.0× 10.0 mm, compressed sensing (CS) factor = 3, partial Fourier (PF) factor = 0.8, temporal resolution = 96 ms, read‐out bandwidth (BW) = 1884 Hz/px) was used. The field‐of‐view (FOV) and in‐plane reconstructed voxel size were 250 × 250 ${\mathrm{mm}}^{2}$ and 2.6 × 2.6 ${\mathrm{mm}}^{2}$ for the coronal bSSFP (c bSSFP) cine images with matrix size 96 × 96. The FOV and in‐plane reconstructed voxel size were 280 × 158 ${\mathrm{mm}}^{2}$ and 2.2 × 2.2 ${\mathrm{mm}}^{2}$ for the sagittal bSSFP (s bSSFP) cine images with matrix size 127 × 71. Second, a $T_{1}$‐spoiled single‐shot gradient echo ($T_{1}$‐GRE) cine sequence (TR/TE = 2.6/1.4 ms, FA = 10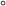, acquisition voxel size = 4.0 × 4.0 × 10.0 ${\mathrm{mm}}^{3}$, CS factor = 3, temporal resolution = 97 ms, BW = 500 Hz/px) was used. The FOVs were 225 × 225 ${\mathrm{mm}}^{2}$ and 225 × 178 ${\mathrm{mm}}^{2}$ in the coronal and sagittal planes with matrix sizes 79 × 79 and 79 × 55 respectively. The coronal and sagittal $T_{1}$‐GRE cine images (c $T_{1}$‐GRE and s $T_{1}$‐GRE) were reconstructed with an in‐plane resolution of 3.2 × 3.2 ${\mathrm{mm}}^{2}$. The $T_{1}$‐GRE sequence was parametrized using larger voxel sizes and lower read‐out BW (with respect to the bSSFP sequence) to bolster the signal‐to‐noise (SNR) ratio. The image‐readout was directed in the head‐feet direction in both bSSFP and $T_{1}$‐GRE sequences. The sequences were adapted to comply to the safety guidelines for scanning patients with a CIED. The safety requirements were met by limiting the specific absorption rate (SAR) to 2.0 W/kg and the time‐varying magnetic field ($\frac{dB}{dt}$) to 80 mT/s.^24^ The SAR and $\frac{dB}{dt}$ values were calculated and displayed by the scanner software.

#### 2.1.1 Phantom cine MRI acquisitions

Single‐slice cine images were acquired using an MR‐compatible Quasar ${\mathrm{MRI}}^{4D}$ (IBA Quasar, London, ON, Canada) motion phantom consisting of a water‐filled oval body with two cylindrical inserts dedicated for a moving and a static component. The body oval was placed on the table in the center of the bore of the 1.5 T Unity MR‐linac for experimentation. A movable cylinder insert consisting of a spherical object with 3 cm diameter was used as target and its center‐point for center‐of‐gravity motion estimation purposes. The cylinder insert was moved according to an artificial cardiorespiratory motion trajectory consisting of cardiac ($\cos^{4}$, 70 bpm, 10 mm peak‐to‐peak) and respiratory (sine, 12 bpm, 20 mm peak‐to‐peak) motion components.^12^

#### 2.1.2 Healthy volunteers cine MRI acquisitions

Single‐slice balanced SSFP and $T_{1}$‐GRE cine images were acquired in free‐breathing in five healthy volunteers with the arms down (3 male, 2 female, mean age ± SD = 27 ± 2.3 years) after obtaining written informed consent (study ID: NL59820.041.17). The cine MRI images were acquired with and without an ICD per volunteer for the investigation of ICD‐induced image quality and geometric fidelity degradation. The ICD‐induced effects on the acquired cine MRI images were investigated by placing an MR‐conditional ICD (Claria MRI, CRT‐D SureScan, Model DTMA2D1, Medtronic Inc., MN, USA) on the clavicle within a protective envelope of water‐equivalent gel. The acquired cine sets consisted of 200 dynamic frames in steady‐state.

#### 2.1.3 VT patient cine MRI acquisitions

A single (from a total of three) VT patient (male, 83 years old) with an implanted ICD (and metallic aortic valve replacement) who received a STAR treatment within the PRO‐STAR trial (study ID: NL76535.041.21) was scanned on a 1.5 T MRI‐simulator (Ingenia, Philips Healthcare, Best, The Netherlands) with the arms up. Two patients were not eligible for MRI scanning due to contra‐indications. The single‐slice cine images were acquired in free‐breathing with the bSSFP (TR/TE = 2.8/1.4 ms, FA = 50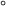, FOV = 325 × 325 ${\mathrm{mm}}^{2}$, acquisition voxel size = 2.8 × 2.8 × 10 ${\mathrm{mm}}^{3}$, SENSE = 3.5, temporal resolution = 114 ms, BW = 1710 Hz/px, 200 frames) and $T_{1}$‐GRE (TR/TE = 2.6/1.3 ms, FA = 10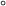, FOV = 325 × 325 ${\mathrm{mm}}^{2}$, acquisition voxel size = 2.8 × 2.8 × 10 ${\mathrm{mm}}^{3}$, SENSE = 2.7, temporal resolution = 149 ms, BW = 1710 Hz/px, 150 frames) sequences. All cine images had a matrix size of 176 × 176. The cine acquisition sequence parameters were differently adjusted with respect to the imaging parameters described in Section 2.1 according to clinical needs at the time.

### 2.2 Cardiorespiratory motion validation in phantom

The obtained phantom cine images were processed offline in Matlab 2023a (Mathworks, Natick, MA, USA) for motion estimation of the target object. The center‐of‐gravity motion of the spherical target object was estimated by using a built‐in circular Hough transform (i.e., image‐based circle detection) on spatially upsampled (5 × using linear interpolation) cine images. The imaging latency between acquiring the center of k‐space ($k_{0}$) and receiving each frame of the proposed cine sequence was measured by extracting the time difference between the phantom‐reported positions and MR image‐derived positions.^25^ The obtained motion traces were then temporally aligned for point‐wise comparisons using the imaging latency. The accuracy of motion estimation using the acquired cine images was quantified using the root‐mean‐squared error (RMSE) using corresponding phantom‐reported position(s) as reference(s), which were reported in real‐time during image acquisition.

### 2.3 Cardiorespiratory motion validation in vivo

**FIGURE 1:**
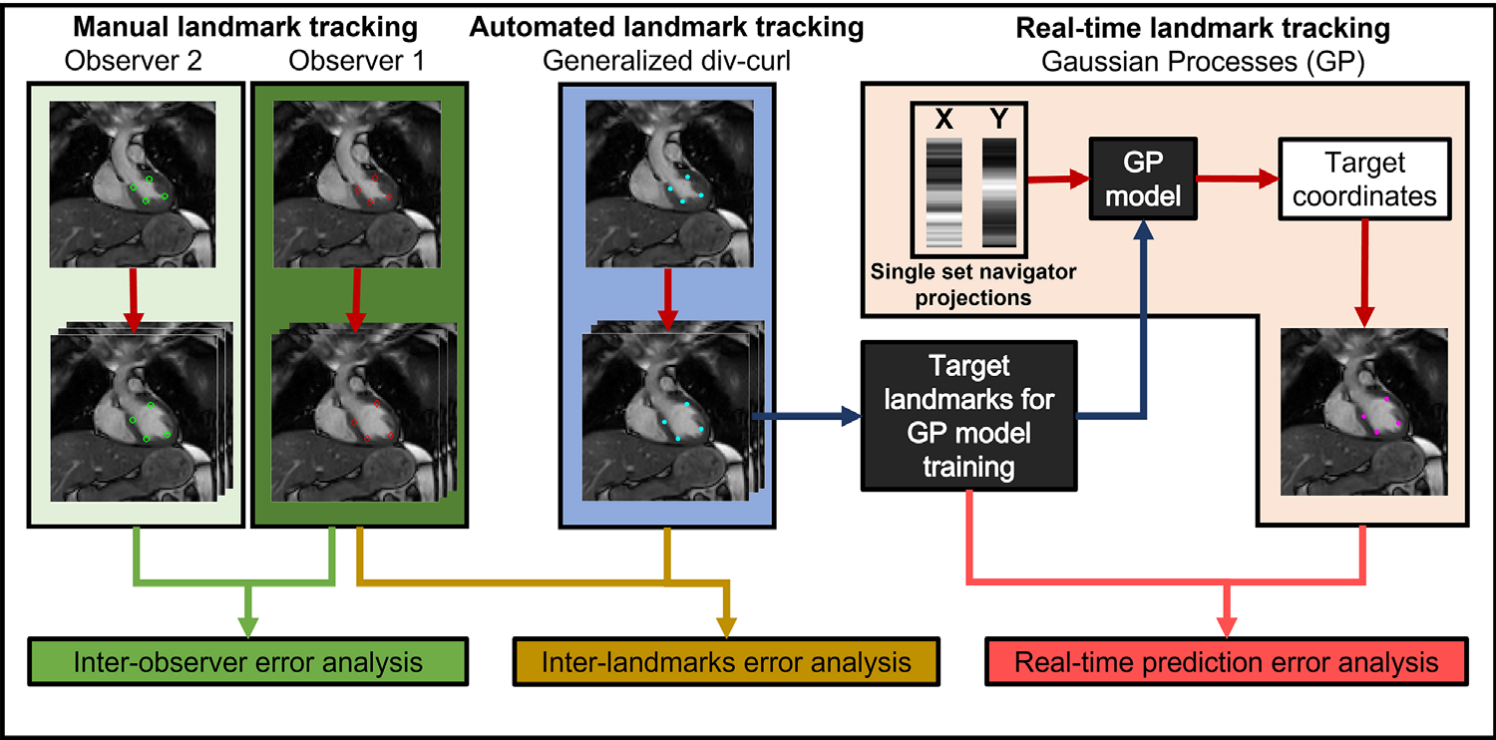
An overview of the three applied methods for in vivo validation of automatic and manual cardiorespiratory motion estimation.

The acquired in vivo cine images were processed offline in Matlab 2023a for retrospective motion estimation of selected myocardial landmarks within the left ventricular myocardial wall. For healthy volunteers, the left ventricle (LV) was divided in four quadrants in which the motion of a single landmark (primarily endocardial) was estimated in each of the quadrants. For the patient, the center‐of‐gravity motion of the gross target volume (GTV) was estimated. An overview of the cardiorespiratory motion estimation process is schematically shown in Figure 1. For validation purposes, the myocardial landmarks were manually annotated by two independent observers (Section 2.3.1), which was labor intensive and time‐consuming. Therefore, the position of the landmarks was estimated with an automated approach by using a generalized divergence‐curl (GDC) DIR algorithm (Section 2.3.2).^26^ For real‐time motion estimation purposes, the position of the myocardial landmarks was also estimated using Gaussian processes (GP) (Section 2.3.3).^27^, ^28^

#### 2.3.1 Manual landmark tracking

For one volunteer dataset, four myocardial landmarks (1 per LV quadrant) were annotated by two independent observers on a series of 200 spatially upsampled (4 × using linear interpolation) dynamic frames (approximately 20 s). The inter‐observer deviation of the manually annotated landmarks was investigated and reported using the absolute point‐wise deviation (PWD) and RMSD metric. Manual in‐plane 2D annotations of a single observer were used subsequently as reference to quantify the accuracy of the automated motion estimation approaches via GDC by reporting the in‐plane 2D PWD (PWD^2D^) and RMSD (RMSD^2D^) between the obtained motion traces. Statistical testing was performed using the two‐sided Wilcoxon signed‐rank test (α=0.05) to establish the significance of the inter‐observer differences of the manually annotated landmarks.

#### 2.3.2 Automated landmark tracking

DIR allows for the estimation of a vector field $\vec{u}$ quantifying motion occurring between a set of a fixed (F) and moving images (M). In order to recover motion that is consistent with the underlying biomechanical tissue properties, we employed the GDC registration model that allows the application of independent penalties on the divergence, curl of their deformations and/or their *n* ^th^ order derivative. In particular, the GDC‐registration model minimizes the functional described with Equation (1).

(1) $$L=\int_{\Omega }D\left(F,M,\vec{u}\right)+\alpha {\left\|\nabla \;\vec{u}\right\|}^{2}+\beta {\left\|\nabla \nabla \;\vec{u}\right\|}^{2}+\gamma {\left\|\nabla \times \;\vec{u}\right\|}^{2}\;d\vec{x}$$

Here, Ω represents the image domain, D represents the similarity metric, which was chosen to be the local correlation coefficient (LCC).^29^

(2) $$\text{LCC}\left\{F,M\right\}\left(\vec{x}\right):=\frac{{\left(\sum_{\vec{z}\in S\left(\vec{x}\right)}\left(F\left(\vec{z}\right)-\bar{F}\right)\cdot \left(M\left(\vec{z}\right)-\bar{M}\right)\right)}^{2}}{\sum_{\vec{z}\in S\left(\vec{x}\right)}{\left(F\left(\vec{z}\right)-\bar{F}\right)}^{2}\cdot \sum_{\vec{z}\in S\left(\vec{x}\right)}{\left(M\left(\vec{z}\right)-\bar{M}\right)}^{2}}$$

The LCC generalizes the cross‐correlation metric by assuming a local linear relationship between image intensities, which is defined according to Equation (2) at any chosen pixel $\vec{x}$. The first regularization term in Equation (1) ensures that the magnitude of the local expansions/contractions is bounded. The second term enforces some degree of spatial smoothness on expansions/contractions. The third term penalizes the magnitude of rotations. The values that we used were α=0.01,β=0.01,γ=16. The regularization weights α,β, and γ were optimized using a single training dataset with 150 manual myocardial landmark annotations.

Here, $S(\vec{x})$ denotes a spherical neighborhood around $\vec{x}$ with a radius of 3 pixels and both $\bar{F}$ and $\bar{M}$ represent the image averages within this neighborhood.

The inter‐landmark deviation was computed by comparing the resulting motion patterns of the myocardial landmarks with the manual myocardial landmark annotations of the first observer. The absolute PWD and RMSD^2D^ scores were extracted for comparison. The point‐wise inter‐landmark deviations were statistically tested on significance using the two‐sided Wilcoxon signed‐rank test (α=0.05).

#### 2.3.3 Real‐time landmark tracking

The feasibility of real‐time cardiorespiratory motion estimation using GP was previously demonstrated without specifically accounting for CIED‐induced artifacts.^30^ In this study, the previously developed GP‐model was retrospectively applied for the evaluation of cardiorespiratory motion estimation performance using the acquired cine images with and without presence of an ICD. The application of GP was a two‐step approach that comprised of subject‐specific GP‐model training and real‐time inference (Figure 1). For the training phase, the GP algorithm required a paired set of k‐space navigator readouts as input with corresponding reference (“ground truth”) values (i.e., myocardial landmark annotations). The internal model parameters were then trained by GP to approximate a function that predicted the motion of the myocardial landmark based on a set of image projections in each orthogonal direction. During the inference phase, the trained GP‐model was applied to estimate the following landmark locations using single sets of image projections. The GP‐model was trained using the GDC‐derived landmarks (first 50% of each cine series). We evaluated the real‐time motion prediction performance of our applied GP‐model by calculating the real‐time prediction error, which was the point‐wise error between the GDC‐derived landmarks and GP‐predicted targets of the last 50% of each cine series.

### 2.4 Static magnetic field‐mapping

The effects of the leads and ICD on the $B_{0}$‐field were investigated by acquiring volumetric $B_{0}$‐maps with a 3D GRE sequence in phantom (cf. Section 2.4.1) and in vivo (Section 2.4.2) on the 1.5 T MR‐linac. Two acquisitions were made with an echo time difference (dTE) of 4.6 ms. The voxel‐wise phase difference between these two acquisitions yielded the $B_{0}$‐map.^31^ The discontinuous $B_{0}$‐map (due to phase wraps) was unwrapped using a region‐growing unwrapping algorithm.^19^

#### 2.4.1 $B_{0}$‐mapping in phantom

The Quasar ${\mathrm{MRI}}^{4D}$ motion phantom was used in an experimental setup to perform $B_{0}$‐mapping in presence of cardiac device leads using a 3D GRE sequence (TR/TE = 11/4.6 ms, FA = 15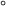, FOV = 308 × 401 × 400 ${\mathrm{mm}}^{3}$, acquisition voxel size = 2 × 2 × 2 ${\mathrm{mm}}^{3}$, SENSE = 1.5 (RL [right–left]), BW = 556 Hz/px) on the 1.5 T MR‐linac. The body oval was placed on the table in the center of the bore of the MR‐linac during the field mapping acquisitions. The cardiac device leads were fixed on a perspex rod in the middle of a water‐filled moving cylinder insert, which was functioning as a scaffold (Figure S2). Three different MR‐conditional leads were included in the phantom experiments (Table 1).

**TABLE 1:** Characteristics of CIED leads used in this study.

	Length (cm)	⌀ (mm)	Type	Vendor	Lead model
Lead 1	58	2.0	Pacing	Medtronic	5076
Lead 2	88	1.3	Pacing	Medtronic	4796
Lead 3	55	2.8	Defibrillation	Medtronic	6947M

The $B_{0}$‐maps were acquired while the cylinder insert was moving according to a pre‐defined artificial cardiorespiratory motion waveform consisting of cardiac ($\cos^{4}$, 60–70 bpm, 10 mm peak‐to‐peak) and respiratory (sin, 12 bpm, 20 mm peak‐to‐peak) motion components.

The magnitude images obtained from the $B_{0}$‐map acquisitions were used to delineate the structures (i.e., cylinder insert, perspex rod and cardiac leads), while the cylinder insert was stationary in the isocenter. The location of the leads were estimated by delineating the volumetric lead‐induced signal void with a range of margins (i.e., [1–5] mm) to quantify the geometric fidelity in shells with varying distances. The delineated structures were used to calculate the frequency offsets within the structures, but also to estimate the size of the lead‐induced signal void in the transversal plane. The frequency offsets were used afterwards to calculate the spatial distortions for our proposed cine sequences (Section 2.1). The mean±SD and the 98th percentile range of the spatial distortions were computed.

#### 2.4.2 $B_{0}$‐mapping in vivo

During the in vivo cine acquisitions, $B_{0}$‐mapping was performed with and without ICD for each volunteer. The static field map was acquired using a 3D GRE sequence in free‐breathing (TR/TE = 11/4.6 ms, FA = 15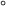, FOV = 308 × 401 × 400 ${\mathrm{mm}}^{3}$, acquisition voxel size = 4 × 4 × 4 ${\mathrm{mm}}^{3}$, SENSE = 1.5 (RL), BW = 556 Hz/px) on the 1.5 T MR‐linac. The effect of the ICD on the $B_{0}$‐field was evaluated by comparing it to the $B_{0}$‐maps, which were acquired without ICD. The estimated frequency offsets and imaging parameters of our proposed cine images were used to convert the frequency offsets to spatial distortions.

A $B_{0}$‐map acquisition sequence was also included during the MRI examination of the VT patient to investigate the CIED‐induced field distortions. The GTV and surrounding OARs were delineated by an experienced radiation oncologist on the volumetric acquisitions within the complete pre‐treatment imaging protocol and were retrospectively warped on the cine images by clinically used DIR algorithms. The geometric fidelity in the patient data was investigated within delineated structures in the heart. The frequency offsets in the GTV, planning target volume (PTV), whole heart (WH) and LV were included for spatial distortion calculations according to the used cine imaging sequence parameters (Section 2.1).

## 3 RESULTS

### 3.1 Motion validation in phantom

The RMSE for the center‐of‐gravity motion with respect to the phantom reference was 0.6 mm in the coronal plane and 0.5 mm in the sagittal plane when estimating the phantom position using our proposed bSSFP cine sequence. For our proposed T_1_‐GRE cine sequence, the RMSE was quantified at 0.9 mm in the coronal plane and 1.0 mm in the sagittal plane. The imaging latency was measured to be 100±2.3 ms for the bSSFP cine sequence and 110±6.5 ms for the T_1_‐GRE sequence. The circle detection is shown on a single frame acquired with every cine sequence with its corresponding quantified motion traces shown in Figure S1.

### 3.2 In vivo experiments

#### 3.2.1 Visual assessment

**FIGURE 2:**
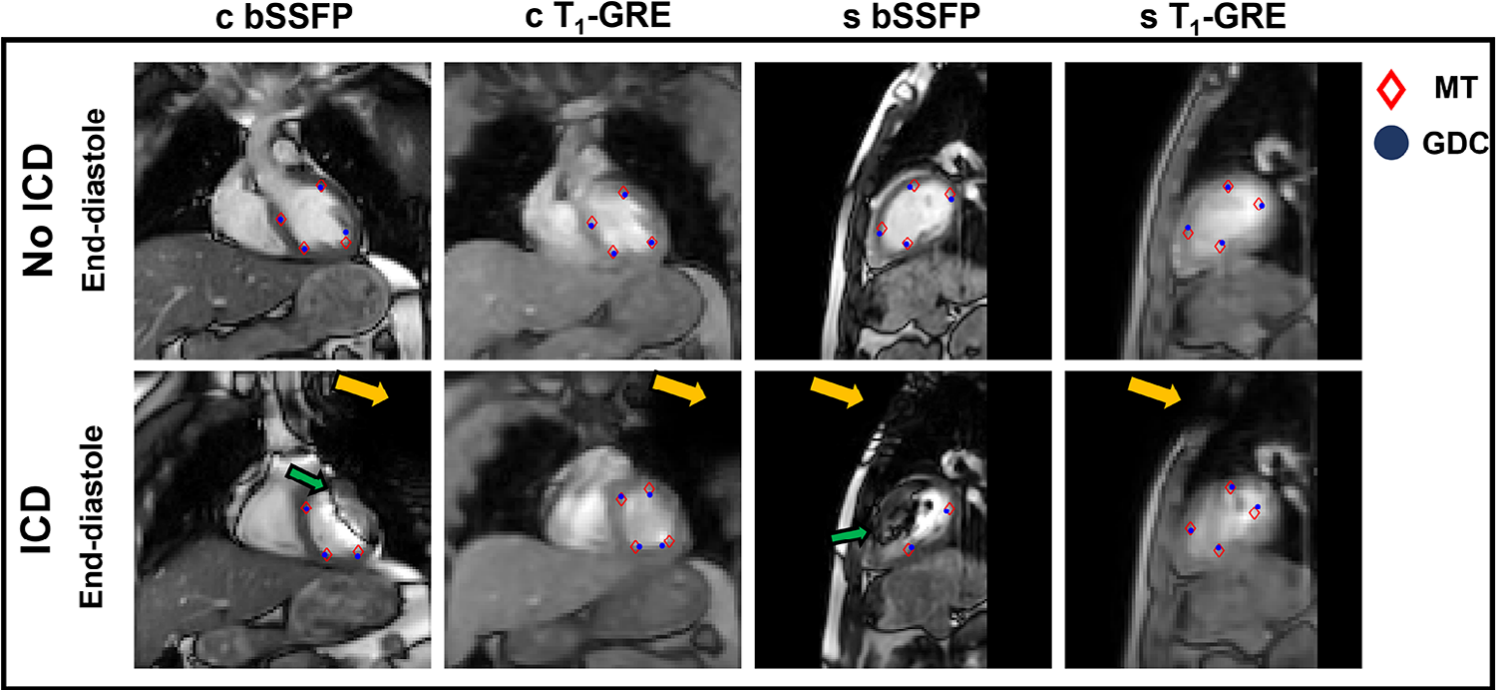
Cine acquisitions from a single healthy volunteer (V5) acquired with and without an ICD. Acquired frames in end‐diastole are shown. The ICD (yellow arrows) was placed on the clavicle within a protective envelope. The green arrows indicate the ICD‐induced banding artifacts in the left ventricle. The annotated myocardial landmarks were used for cardiorespiratory motion estimation using MT of the first observer and GDC deformable image registration. ICD, implantable cardioverter–defibrillator; GDC, generalized divergence‐curl; MT, manual tracking.

Single frames of the acquired cine acquisitions are shown in Figure 2. Here, the variation in image quality between the sequences is apparent. The contrast between the blood pool and the myocardium is reduced on the T_1_‐GRE cine images with respect to the bSSFP cine images. The cine images in presence of an ICD (which is indicated by the yellow arrow on the sagittal and coronal images) are severely disrupted in the bSSFP images. The LV is visually unaffected in the T_1_‐GRE cine images as the ICD‐induced artefacts are limited to a signal void proximal to the LV.

**FIGURE 3:**
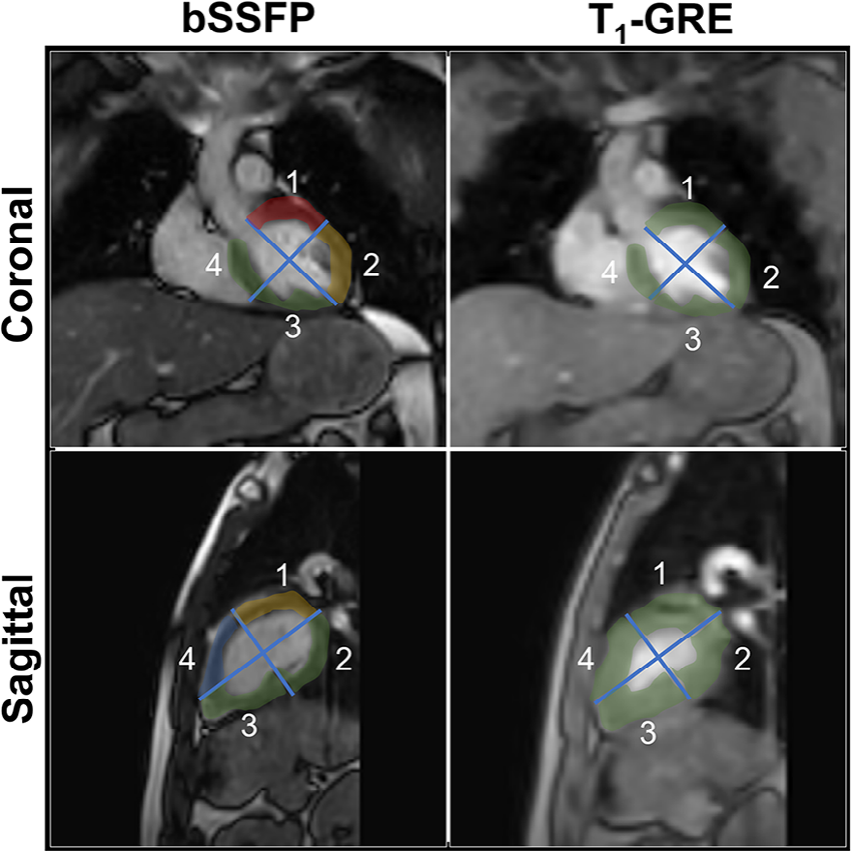
An overview of the left ventricular quadrants depicted on a single frame of every used cine sequence with corresponding color overlay indicating the success rate of cardiorespiratory motion estimation. Green indicates a 100% success rate, yellow 80%, blue 60%, and red 40%.

Cardiorespiratory motion was estimated in 85% of the LV quadrants using the bSSFP sequence and in 100% of the LV quadrants using the T_1_‐GRE sequence after visually assessing the cine images in all volunteer datasets. In Figure 3, the distribution of included LV quadrants for cardiorespiratory motion estimation is shown. The feasibility of motion estimation of myocardial landmarks was mainly restricted in quadrant 1 in the coronal plane and quadrants 1 and 4 in the sagittal plane.

**FIGURE 4:**
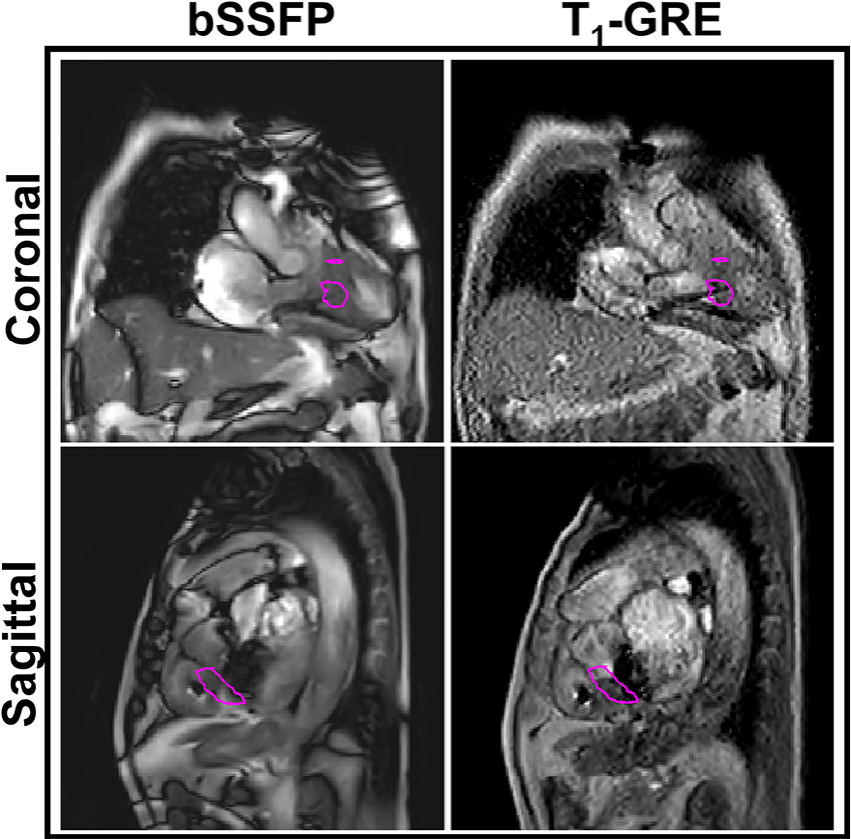
Single cine frames acquired in patient with delineations of main cardiac structures in the heart and GTV. GTV, gross‐target‐volume.

Single frames of the acquired cine images from the VT patient are shown in Figure 4. The bSSFP cine images demonstrated good contrast between the myocardium and the blood pool with respect to the T_1_‐GRE cine images. The arms of the VT patient were positioned up and effectively increased the distance of the ICD from the heart. Consequentially, the banding artifacts on the bSSFP cine images did not affect the visualization of the LV. The aortic valve replacement and cardiac device leads induced local artifacts, but did not obscure the GTV. The T_1_‐GRE cine images have lower SNR and contrast with respect to the bSSFP images, but demonstrated its robustness against the CIED‐induced artifacts.

#### 3.2.2 Motion estimation

**FIGURE 5:**
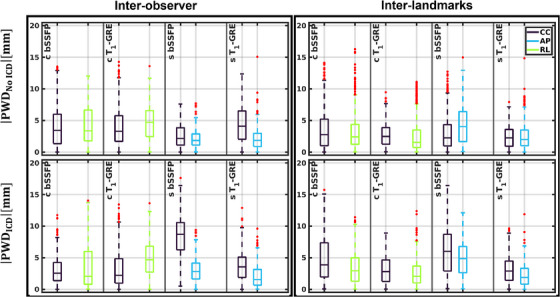
Left: absolute point‐wise errors between the annotated myocardial landmarks by two observers. A total of 1.7% and 0.7% of the datapoints were considered outliers with and without ICD, respectively. Right: absolute point‐wise errors between the manually annotated myocardial landmark by the first observer versus the estimated myocardial landmarks using the GDC deformable image registration algorithm. A total of 0.8% and 2.7% of the datapoints were considered outliers with and without ICD, respectively. Data points were considered outliers when they exceed 1.5 times the inter‐quartile range. ICD, implantable cardioverter– defibrillator; GDC, generalized divergence‐curl.

The inter‐observer and inter‐landmarks PWD of all four myocardial landmarks between the manually annotated myocardial landmarks were calculated in the cranial–caudal (CC), anterior–posterior (AP), and RL directions and shown with boxplots in Figure 5. The in‐plane 2D motion extents (i.e., Euclidean peak‐to‐peak motion) ranged between 13.3–35.2, 13.3–37.8, and 8.3–22.2 mm estimated by the first observer, second observer and GDC‐DIR, respectively. Without ICD, the inter‐observer RMSD^2D^ was 2.8 mm in the coronal plane and 2.7 mm in the sagittal plane using the bSSFP sequence. Using the T_1_‐GRE sequence, the RMSD^2D^ was 4.5 mm in the coronal plane and 3.7 mm in the sagittal plane. With ICD, the RMSD^2D^ was 3.9 mm in the coronal plane and 5.6 mm in the sagittal plane using the bSSFP sequence. The RMSD^2D^ was 3.1 mm in the coronal plane and 3.2 mm in the sagittal plane using the T_1_‐GRE sequence. Two‐sided Wilcoxon signed‐rank tests showed that the inter‐observer errors using the coronal T_1_‐GRE (*p* = 0.4) and sagittal bSSFP (*p* = 0.63) datasets were not significant.

The computation time of the GDC‐algorithm was $O(1\cdot 10^{1})$ s per cine frame. The RMSE^2D^ between the GDC‐model‐derived landmarks and the manually annotated landmarks (both visualized in Figure 2) of a single observer ranged between 3.1 and 4.1 mm across the single‐used healthy volunteer dataset (V5) without ICD. With ICD, the RMSD^2D^ ranged between 3.2 and 4.6 mm. Two‐sided Wilcoxon signed‐rank tests showed that the observer and GDC algorithm differed significantly (*p* = 0.00).

**FIGURE 6:**
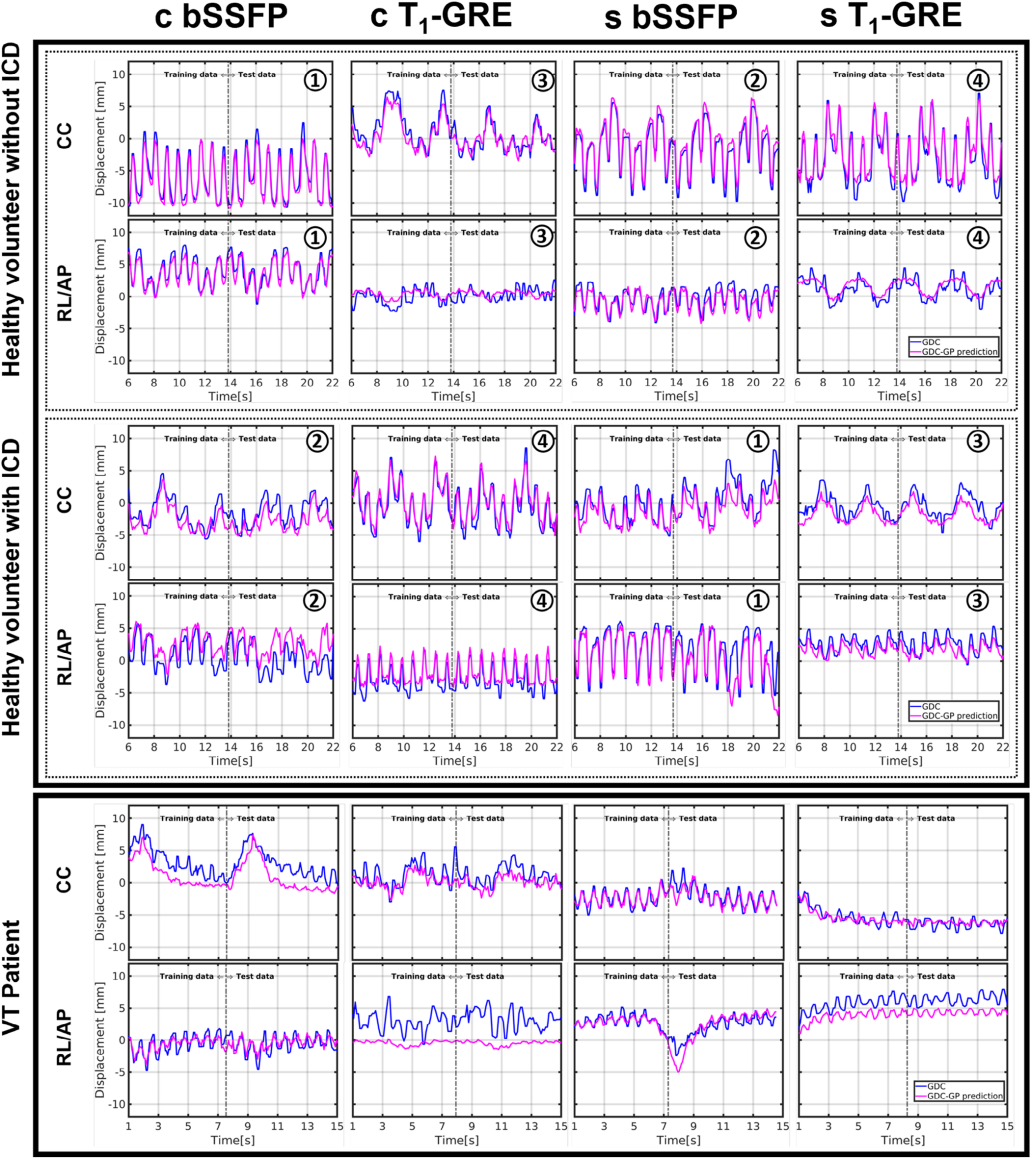
An overview of selected cardiorespiratory motion traces at varying positions in the left ventricle of volunteer 5 (with and without ICD). The number in the top right corner of each graph corresponds to the LV quadrant in the healthy volunteer. The motion traces of the VT patient were estimated in the GTV center. The motion traces were estimated using GDC and GP. ICD, implantable cardioverter– defibrillator; GDC, generalized divergence‐curl; GP, Gaussian processes; GTV, gross target volume; LV, left ventricle; VT, ventricular tachycardia.

**FIGURE 7:**
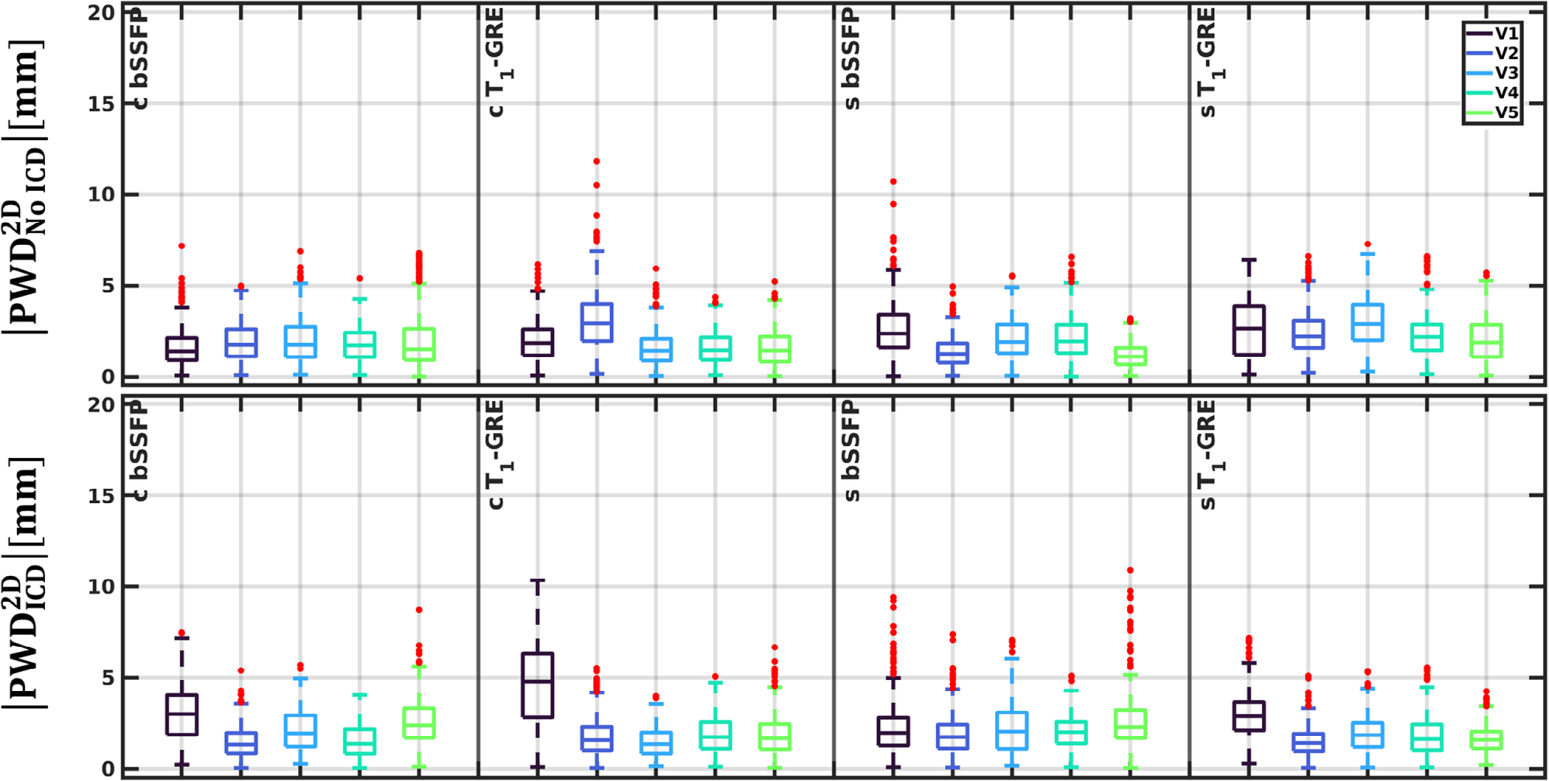
The absolute in‐plane point‐wise difference (PWD^2D^) per sequence for all volunteer datasets between the predicted positions using Gaussian processes and the reference data using the generalized div‐curl estimated myocardial positions. Data points are considered outliers when they exceed 1.5 times the inter‐quartile range.

With 200 dynamics, the time required to train the GP‐model was $O(1\cdot 10^{0})$ s, while the inference time was $O(1\cdot 10^{-2})$ s. A selection of motion traces of various myocardial landmarks are displayed in Figure 6. The deviations between the GP‐predictions and GDC‐derived landmarks are shown using a more condensed approach by displaying the boxplots of in‐plane 2D PWD (PWD^2D^) in Figure 7. Without ICD, the RMSD^2D^ between the GP‐predictions and GDC‐derived annotations ranged between 0.9 and 2.2 mm using the bSSFP sequence. With the T_1_‐GRE sequence, the RMSD^2D^ ranged between 1.3 and 3.0 mm. In presence of an ICD, the RMSD^2D^ ranged between 1.3 and 2.2 mm using the bSSFP sequence, while the RMSD^2D^ ranged between 1.2 and 3.2 mm.

Center‐of‐gravity cardiorespiratory motion of the GTV in the VT patient was estimated and (a part of) the motion traces are shown in Figure 6. The RMSD^2D^ between the GDC‐model‐derived landmarks and GP‐predictions was 1.6 mm in the coronal plane and 1.2 mm in the sagittal plane using the bSSFP sequence, while the RMSD^2D^ was 3.1 mm in the coronal plane and 1.8 mm in the sagittal plane using the T_1_‐GRE sequence.

### 3.3 Static magnetic field mapping

#### 3.3.1 Field mapping in phantom

The 98^th^ percentile range of the measured frequency offsets decreased (measured from 1 to 5 mm margins) with a maximum of 36.5%, which was observed in lead 3. Using the bSSFP sequence, the expected mean lead‐induced spatial distortions were in the range of 0.0 to 0.1 mm. With the T_1_‐GRE sequence, the expected mean lead‐induced spatial distortion varied between 0.1 and 0.3 mm. A single slice of the magnitude, wrapped and unwrapped $B_{0}$‐maps with lead 1 in each orthogonal plane is shown in Figure S3 in which the lead‐induced signal void and lead shells are depicted within the movable cylinder insert. Similar depictions are shown in Figures S4 (lead 2) and S5 (lead 3). The trend of the mean frequency offsets and 98^th^ percentile range per lead shell of each lead is depicted in Figure S6 with the corresponding expected spatial distortions based on our used cine sequence parameters. The actual values are summarized in Table S1.

#### 3.3.2 Field mapping in vivo

The presence of the ICD increased the absolute mean frequency offset in 3 out of 5 volunteer datasets. The 98^th^ percentile range of frequency offsets increased in all volunteer‐derived field maps with ICD with respect to without an ICD between 79.5% and 123.8%.

**FIGURE 8:**
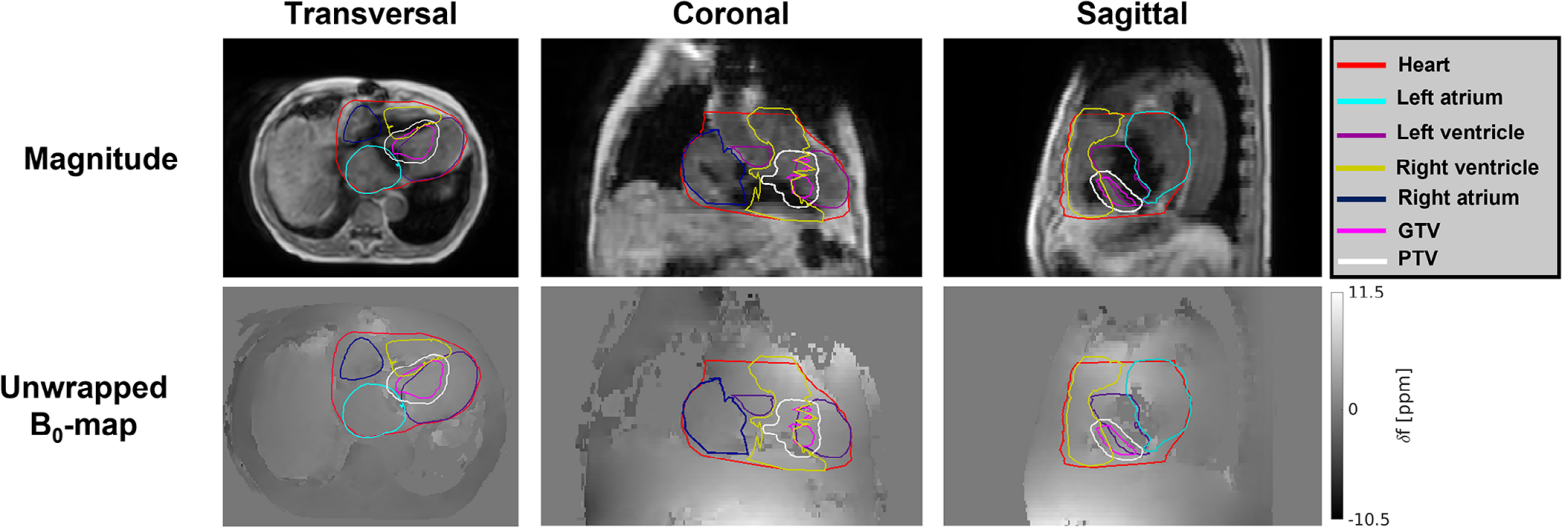
A single slice of the magnitude, phase and unwrapped field map in every orthogonal imaging plane of the patient including the delineations of main cardiac compartments and target volumes.

**FIGURE 9:**
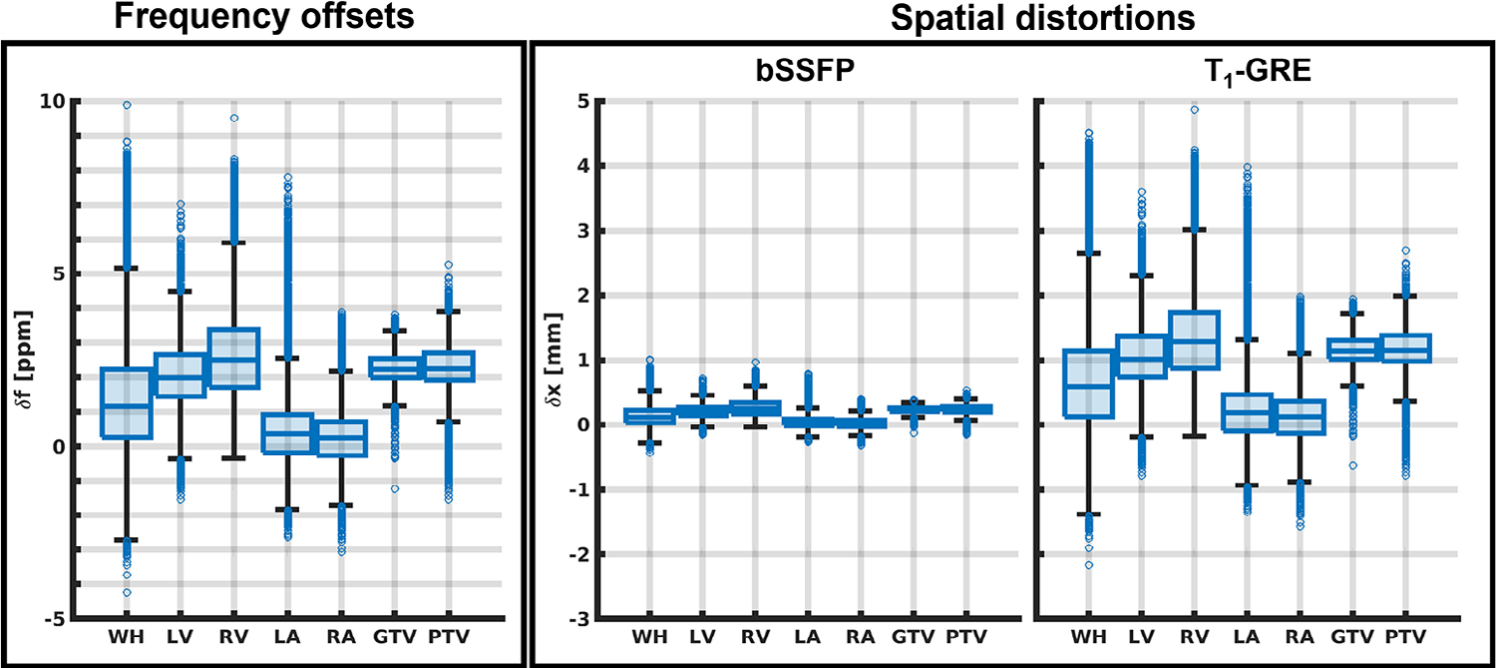
Boxplots giving an overview of the measured frequency offsets in the VT patient data within the WH, LV, RV, LA, RA, GTV and PTV. The measured frequency offsets were used to estimate the expected spatial distortions within the structures using the imaging parameters of our proposed cine imaging sequences. Data points are considered outliers when they exceed 1.5 times the inter‐quartile range. GTV, gross target volume; LA, left atrium; LV, left ventricle; PTV, planning target volume; RA, right atrium; RV, right ventricle; WH, whole heart.

The unwrapped $B_{0}$‐map of the VT patient is shown in Figure 8. The frequency offsets and corresponding expected spatial distortions are visualized with boxplots in Figure 9. The 98^th^ percentile range of the spatial distortion within the GTV was measured to be between 0.0–0.4 and 0.0–1.8 mm using our proposed bSSFP and T_1_‐GRE sequence, respectively. The WH, which is the largest delineated structure, encompassed a wider range of CIED‐induced $B_{0}$‐offsets with resulting 98^th^ percentile range of spatial distortions between −0.1–0.6 and −0.7–3.2 mm using the bSSFP and T_1_‐GRE sequence.

Single slices of the unwrapped $B_{0}$‐maps (with and without presence of an ICD) are shown for each single volunteer dataset in every orthogonal orientation in Figures S7–S11 with the corresponding histogram of estimated frequency offsets in the body contour. The frequency offsets measured in all volunteer datasets are summarized in Table S2 with expected spatial distortions for each used cine acquisition sequence.

## 4 DISCUSSION

In this study, clinically available single‐slice bSSFP and T_1_‐GRE cine MRI acquisition sequences were adapted for cardiorespiratory motion estimation applications on an MR‐linac, while complying to imaging safety guidelines for MRI examinations in presence of a CIED. An automated data processing cascade was developed using GDC‐DIR for automated myocardial landmark tracking which then served as input for training a GP model for real‐time motion predictions. The feasibility of cardiac motion monitoring in a radiotherapy setting using the bSSFP and T_1_‐GRE sequences was extensively evaluated in terms of image quality, cardiorespiratory motion estimation and geometric fidelity in presence of CIEDs.

The bSSFP imaging sequence is the gold standard cine MRI sequence for cardiac imaging applications due to its superior soft‐tissue contrast. In presence of a CIED, however, the sequence suffers from typical banding artefacts that obscure the LV either partially or completely. The T_1_‐GRE sequence was posed as an alternative for cardiac cine MRI due to its robustness against CIED‐induced artefacts, while allowing cardiorespiratory motion estimation and visualization of the myocardium in the LV. The myocardial wall was visualized with sufficient image quality using the T_1_‐GRE sequence with a larger voxel size and similar T_scan_, but the image quality was degraded with respect to the bSSFP sequence in terms of SNR and contrast due to the image parameter trade‐offs.

Validating cardiorespiratory motion estimation in vivo was more challenging compared to the phantom setup due to the lack of reference motion traces. To overcome this limitation, manual annotations were marked by an observer on a single volunteer dataset and compared to the manual annotations of a second observer returning RMSD^2D^ scores between 2.7 and 5.6 mm. The RMSD^2D^ magnitude range indicated that annotating the (same) myocardial landmarks was challenging due to the large deformations of the heart. For automated cardiorespiratory motion estimation purposes, the GDC‐based DIR algorithm was used. This method was optimized based on the manual annotations of the first observer combined with visual checks. The resulting inter‐landmark RMSD^2D^ scores ranged between 3.2 and 4.6 mm, indicating that the inter‐landmark agreement was comparable to inter‐observer agreement.

The GDC‐based DIR method was implementation‐wise a computationally expensive approach that would not be suitable for real‐time motion estimation. The registration of a single dynamic took a couple of seconds which was prolonged, depending on the number of required dynamics, to a couple of minutes. Therefore, the real‐time GP method was used and evaluated. The GP‐model is a machine learning‐based tool and requires training before application. The GDC‐derived targets were used to train the GP‐model and it was able to predict positions with sub‐pixel accuracy (with a single exception among all healthy volunteer datasets), while only requiring single navigator projections from the acquired cine datasets. Given the short amount of time ($O(1\cdot 10^{0})$ s) needed to train the GP‐model and its inference capabilities, it is a powerful tool to use for real‐time applications (e.g., respiratory MLC‐tracking and cardiac beam gating).^12^

The effect of CIEDs on the geometric fidelity during cine imaging was also investigated in this study. The phantom measurements showed that the expected lead‐induced spatial geometry distortions were small and only took effect locally in very close proximity of the signal void caused by the lead. Field mapping in healthy volunteers showed that the presence of an ICD had a prominent effect on the magnetic field (mainly dominant in the thoracic cage region) and deteriorated the geometric fidelity. The patient data has shown that the 98^th^ percentile range of the spatial uncertainty in the GTV ranged between 0.0 and 1.8 mm when using the T_1_‐GRE sequence, but remained sub‐millimeter when using the bSSFP sequence due to its substantially higher BW.

In this study, the cine image acquisitions sequences were tailored according to safety guidelines for imaging in subjects with a CIED and the requirement to achieve a minimum frame rate of 10 Hz for real‐time cardiorespiratory motion estimation. To meet these requirements, parameter trade‐offs had to be made to attain sufficient image quality. The voxel size was set to 3.0 × 3.0 × 10 mm^3^ for the bSSFP sequence and 4.0 × 4.0 × 10 mm^3^ for the T_1_‐GRE sequence. For typical cardiac motion (with motion amplitudes ranging from 0.8 to 16.9 mm),^32^, ^33^, ^34^ the motion estimation can be challenging due to the low amount of voxels encompassing the cardiac motion envelope. Additionally, the large slice thickness ensured that the signal of the moving structures was easily captured within the imaging plane during cine MR image acquisitions, which hampered the GDC‐DIR algorithm due to the appearance of myocardial structures within the acquisition plane during contraction and contributed to underestimation of in‐plane motion. The signal in the resulting T_1_‐GRE cine images was improved during protocol optimization by decreasing the read‐out BW. A low read‐out BW strategy is favorable in terms of image quality, but the geometric fidelity becomes lower and would require an adjustment in treatment margins during treatment planning. For future sequence developments, an increased read‐out BW could be more prioritized to decrease CIED‐induced spatial distortions.^16^

In this study, we estimated cardiorespiratory motion in CC, AP, and RL separately in a retrospective fashion using GDC‐based DIR and GP. The automated motion estimation approach was tested and validated on relatively small amount of cine data (with a duration of approximately 20 s) as a proof‐of‐concept. The performance of our automated landmark tracking approach should, however, be tested on much longer cine image acquisitions comparable with the treatment duration of a STAR treatment to test its robustness.

For real‐time and/or online applications, image sequences should be developed that are able to attain 3D cardiorespiratory motion with sufficient spatial and temporal resolution. In recent research, a 16‐fold accelerated image acquisition sequence was developed by combining CS with a deep‐learning model that enabled real‐time cardiac cine imaging with a spatial resolution of 1.8 × 1.8 × 1.8 mm^3^ and temporal resolution between 28 and 34 ms.^35^ If a real‐time 3D cardiac cine MRI sequence is available, our GDC‐based DIR algorithm could be expanded to 3D for automated landmark tracking. The landmarks determined by the GDC‐based DIR algorithm could then be used to train a GP‐model for real‐time applications.^28^ In this study, the GDC‐based DIR was deployed with the assumption that the in‐plane motion was non‐rotational (hence the high penalty on the curl magnitude) to cope with through plane motion. In 3D, this assumption may not hold as the heart also has a twisting motion behavior during contraction and relaxation. The exact parametrization of the GDC‐DIR algorithm for 3D cardiac data is subject to future research.

Field mapping was done during the imaging experiments to test the geometric fidelity in presence of cardiac devices. We investigated the geometric distortion induced by cardiac device leads with phantom experiments. The lead‐induced distortions were investigated as function of the distance to the signal void caused by the lead, as the lead was not directly visible. During the in vivo experiments, a single MR‐conditional ICD that was available to the investigators was used even though a wide variety of ICDs are available on the market. While the amount of CIEDs was limited, similar artifacts can be expected when using the developed cine sequences in presence of other CIED models. The development of the first MR‐conditional ICD was announced in 2011, suggesting that many potential patients still carry an ICD that is not labeled as MR‐conditional. While non MR‐conditional CIEDs do not directly demonstrate risks during MR examinations,^36^ certain safety guideline should be followed.^37^, ^38^ During our imaging experiments, we complied with MR imaging safety guidelines applicable to any mainstream CIED on the market based on the SAR and $\frac{dB}{dt}$ values returned by the scanner software.^14^, ^15^ These returned values are based on acquisition parameters and patient details (i.e., weight) under average conditions, but the risk for increased local effects (e.g., locally increased heating) could still be present. Older non MR‐conditional CIEDs might have a higher amount of ferromagnetic materials that could also induce greater spatial distortions.

This study focused on the application of real‐time cine MRI acquisitions for online adaptive STAR applications on the MR‐linac in presence of CIEDs. For clinical feasibility purposes, sophisticated pre‐treatment and treatment protocols should be developed specifically for VT patients with CIEDs in which MRI, dosimetric and safety aspects are interrogated.^14^, ^15^, ^39^ Yang et al. have treated four patients with CIEDs on a 1.5 T MR‐linac with treatment targets in the abdominal and pelvic sites by developing their own local safety protocol interrogating MR safety and calculated dose levels in the CIED with positive experience and promising results.^20^ A similar protocol should be developed and expanded for patients with treatment targets in the cardiothoracic region. In our previous work, we installed ECG‐hardware equipment on the MR‐linac that could be used for safety and monitoring purposes.^40^ The development of (pre‐)treatment protocols was, however, outside the scope of this paper and future research should be conducted for clinical implementation purposes.

## 5 CONCLUSION

In this work, bSSFP and T_1_‐GRE cine MRI sequences were used for cardiorespiratory motion estimation using GDC‐DIR and GP in presence of CIEDs. This automated landmark tracking approach yielded comparable results with manual landmark annotations. For real‐time tracking applications, GDC‐DIR is suitable to train a GP model, which has a short inference time ($O(1\cdot 10^{-2}$ s)) time. The bSSFP sequence is prone to CIED‐induced artefacts, which makes it challenging to visualize the desired myocardial target(s) and estimate its motion. The T_1_‐GRE cine image sequence is a viable alternative for such scenario as it is robust against CIED‐induced artefacts and therefore able to facilitate myocardial motion estimation for real‐time applications in case the myocardial target would be obscured using the bSSFP sequence.

## CONFLICT OF INTEREST STATEMENT

The authors declare no conflicts of interest.

## Supporting information

Supporting Information
