# Supplementary material for: Evaluation of the impact of cardiac implantable electronic devices on cine MRI for real‐time adaptive cardiac radioablation on a 1.5 T MR‐linac
Source: Med Phys. 2024 Oct 4;52(1):99–112. doi: 10.1002/mp.17438 (PMC11700006; doi:10.1002/mp.17438)
Supplement: Supplementary file 1 — Supporting Information [file MP-52-99-s001.pdf]

Supplementary material

Cardiorespiratory motion validation in phantom

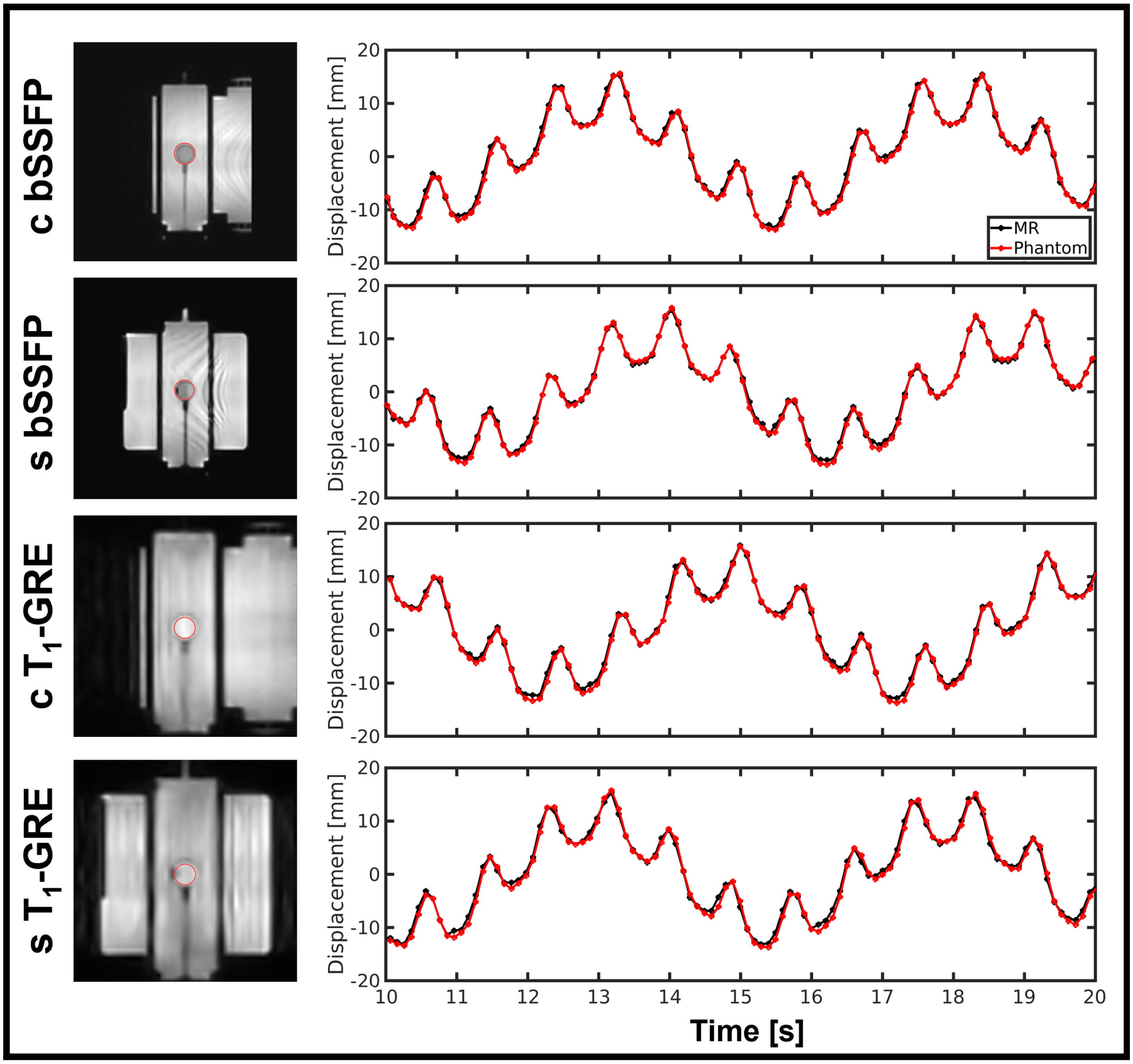

Figure S-1: The extracted center-of-gravity motion of the spherical target using both bSSFP and T_1-GRE sequences in the coronal and sagittal planes and the comparison with the phantom reference are shown.

Phantom B_0-mapping

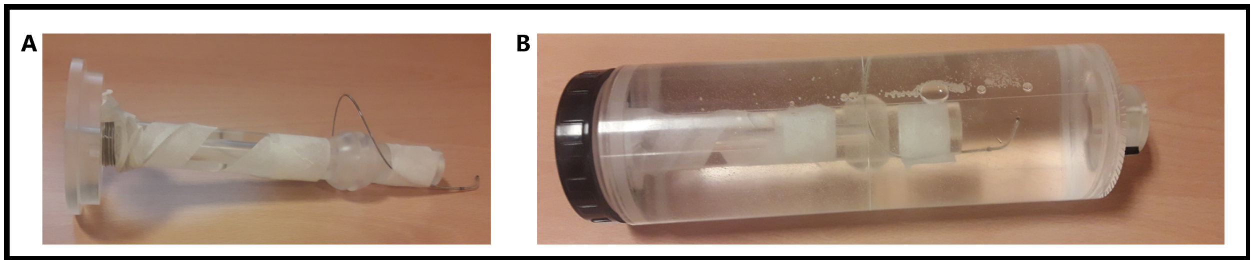

Figure S-2: Demonstration of the cardiac device lead (lead 2) setup attached on the perspex rod insert (c.f., **A**) for the movable cylinder insert (c.f., **B**) of the motion phantom for field mapping. The experimental setup using leads 1 and 3 was similarly prepared.

Phantom B_0 field maps

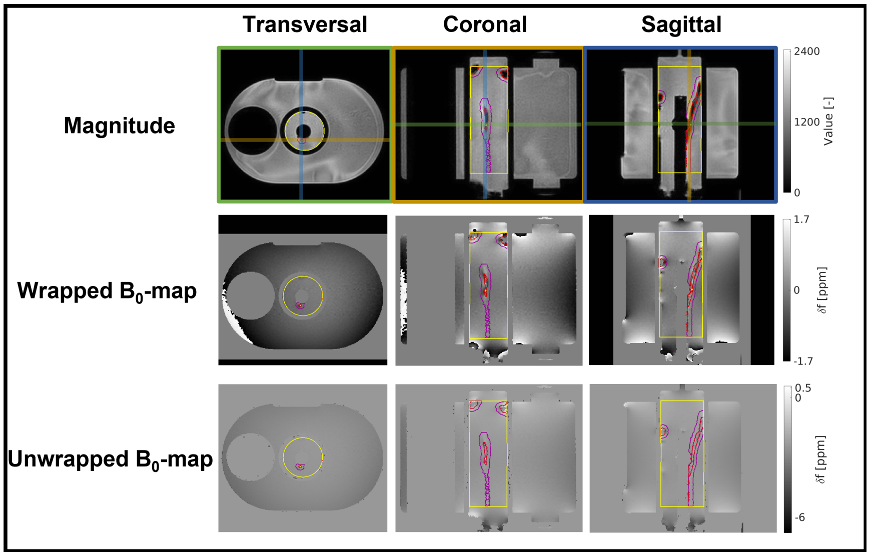

Figure S-3: A single slice in each orthogonal imaging plane of the acquired B_0-map in the motion phantom with lead 1 is shown with the corresponding magnitude image and unwrapped B_0-map. The delineations of lead 1 with margins up to 5 mm with 1 mm increment are visualized.

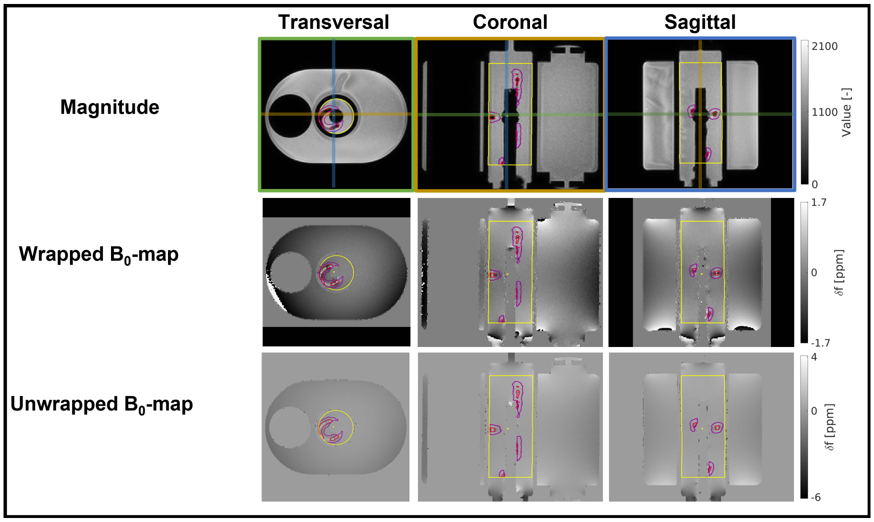

Figure S-4: A single slice in each orthogonal imaging plane of the acquired B_0-map in the motion phantom with lead 2 is shown with the corresponding magnitude image and unwrapped B_0-map. The delineations with margins up to 5 mm with 1 mm increment are visualized.

Table S-1: Field distortion metrics ($\mathbf{f}$) extracted from the phantom experiments with cardiac leads and expected geometric distortions ($\mathbf{x}$) for our bSSFP and T_1-GRE cine sequences (reported as 1st percentile|mean±standard deviation|99th percentile).

	$\mathbf{f}$ [ppm]	$\mathbf{x}_{\text{bSSFP}}$ [mm]	$\mathbf{x}_{\text{T}_1\text{-GRE}}$ [mm]
**Lead 1**			
1 mm	-0.70|0.60±0.58|2.40	-0.07|0.06±0.06|0.24	-0.36|0.31±0.30|1.23
2 mm	-0.61|0.46±0.59|2.31	-0.06|0.05±0.06|0.24	-0.31|0.24±0.30|1.18
3 mm	-0.71|0.49±0.57|2.31	-0.07|0.05±0.06|0.24	-0.36|0.25±0.29|1.18
4 mm	-0.70|0.45±0.56|2.22	-0.07|0.05±0.06|0.23	-0.36|0.23±0.28|1.13
5 mm	-0.53|0.43±0.48|2.02	-0.05|0.04±0.05|0.21	-0.27|0.22±0.25|1.03
**Lead 2**			
1 mm	-0.29|0.19±0.29|0.93	-0.03|0.02±0.03|0.09	-0.15|0.10±0.15|0.48
2 mm	-0.33|0.16±0.28|0.86	-0.03|0.02±0.03|0.09	-0.17|0.08±0.14|0.44
3 mm	-0.34|0.16±0.28|0.82	-0.04|0.02±0.03|0.08	-0.18|0.08±0.14|0.42
4 mm	-0.28|0.19±0.27|0.82	-0.03|0.02±0.03|0.08	-0.14|0.10±0.14|0.42
5 mm	-0.30|0.15±0.28|0.84	-0.03|0.02±0.03|0.09	-0.15|0.08±0.14|0.43
**Lead 3**			
1 mm	-1.46|0.33±0.57|1.83	-0.15|0.03±0.06|0.19	-0.74|0.17±0.29|0.94
2 mm	-1.30|0.36±0.50|1.74	-0.13|0.04±0.05|0.18	-0.67|0.18±0.25|0.89
3 mm	-1.20|0.25±0.47|1.51	-0.12|0.03±0.05|0.15	-0.61|0.13±0.24|0.77
4 mm	-1.08|0.19±0.40|1.18	-0.11|0.02±0.04|0.12	-0.55|0.10±0.21|0.61
5 mm	-0.84|0.19±0.37|1.25	-0.09|0.02±0.04|0.13	-0.43|0.10±0.19|0.64

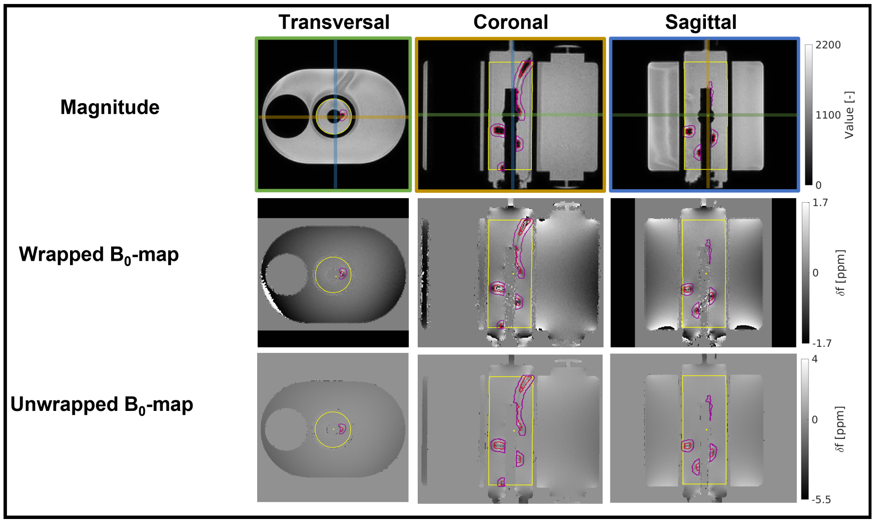

Figure S-5: A single slice in each orthogonal imaging plane of the acquired B_0-map in the motion phantom with lead 3 is shown with the corresponding magnitude image and unwrapped B_0-map. The delineations of lead 3 with margins up to 5 mm with 1 mm increment are visualized.

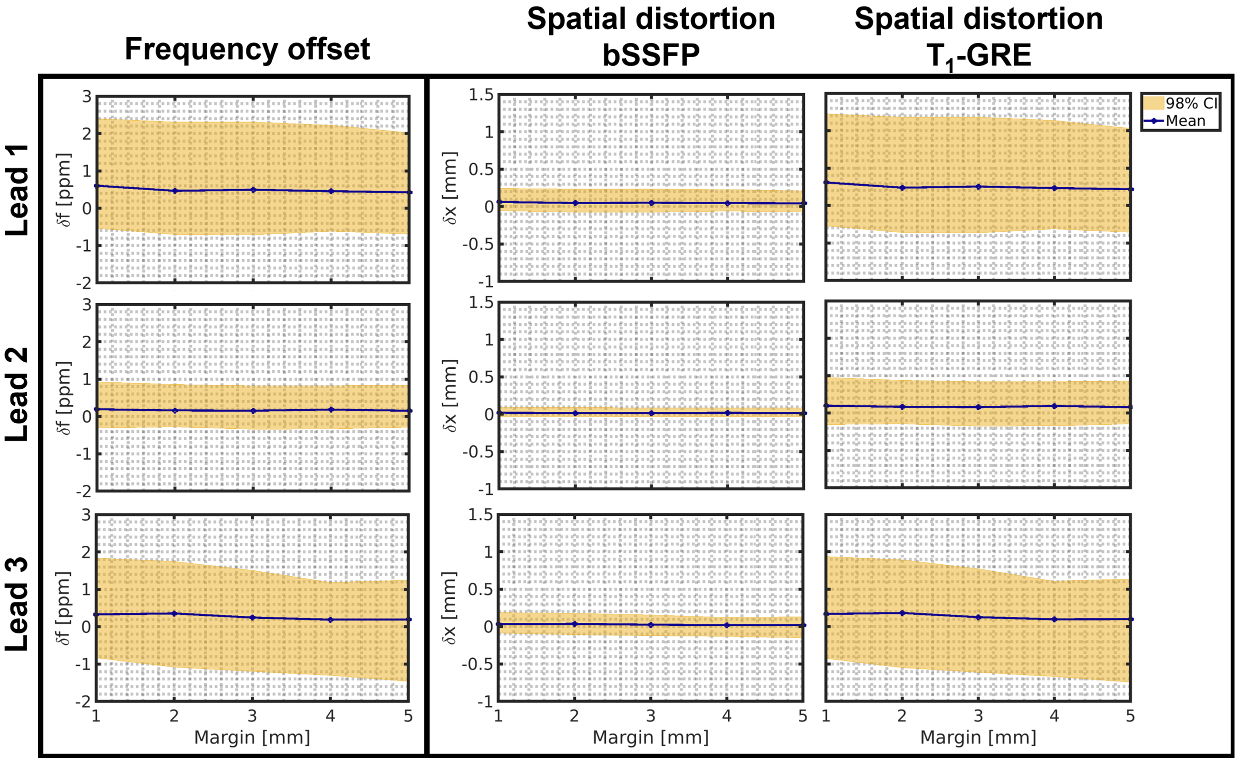

Figure S-6: An overview of the measured frequency offsets (mean and 98th confidence interval (CI)) in phantom in presence of each cardiac device lead with the corresponding expected spatial distortions based on our cine sequence parameters.

Healthy volunteer field maps

Table S-2: Field distortion metrics ($\mathbf{f}$ in ppm) extracted unwrapped in-vivo B_0-maps within the complete body contour with the expected geometric distortions ($\mathbf{x}$ in mm) for our bSSFP and T_1-GRE cine sequences (reported as 1st percentile|mean±standard deviation|99th percentile).

	**f [ppm]**	$\mathbf{x}_{\mathrm{bSSFP}}$ **[mm]**	$\mathbf{x}_{\mathrm{T_1\text{-}GRE}}$ **[mm]**
**V1**			
No ICD	-4.27|0.70±1.62|4.79	-0.43|0.07±0.16|0.49	-2.18|0.36±0.83|2.45
ICD	-6.43|0.57±2.78|9.83	-0.65|0.06±0.28|1.00	-3.29|0.29±1.42|5.02
**V2**			
No ICD	-4.14|0.58±1.40|4.12	-0.42|0.06±0.14|0.42	-2.12|0.30±0.72|2.11
ICD	-8.21|-1.09±2.80|7.90	-0.84|0.11±0.29|0.80	-4.20|-0.56±1.43|4.04
**V3**			
No ICD	-3.86|0.70±1.76|5.70	-0.39|0.07±0.18|0.58	-1.97|0.36±0.90|2.91
ICD	-4.87|2.60±3.50|14.84	-0.50|0.26±0.36|1.51	-2.49|1.33±1.79|7.58
**V4**			
No ICD	-3.39|0.72±1.56|5.23	-0.34|0.07±0.16|0.53	-1.73|0.37±0.80|2.67
ICD	-13.04|-3.27±3.29|6.25	-1.33|-0.33±0.33|0.64	-6.66|-1.67±1.68|3.19
**V5**			
No ICD	-3.45|0.67±1.46|4.76	-0.35|0.07±0.15|0.48	-1.76|0.34±0.75|2.43
ICD	-8.38|0.31±2.91|6.73	-0.85|0.03±0.30|0.68	-4.28|0.16±1.49|3.44

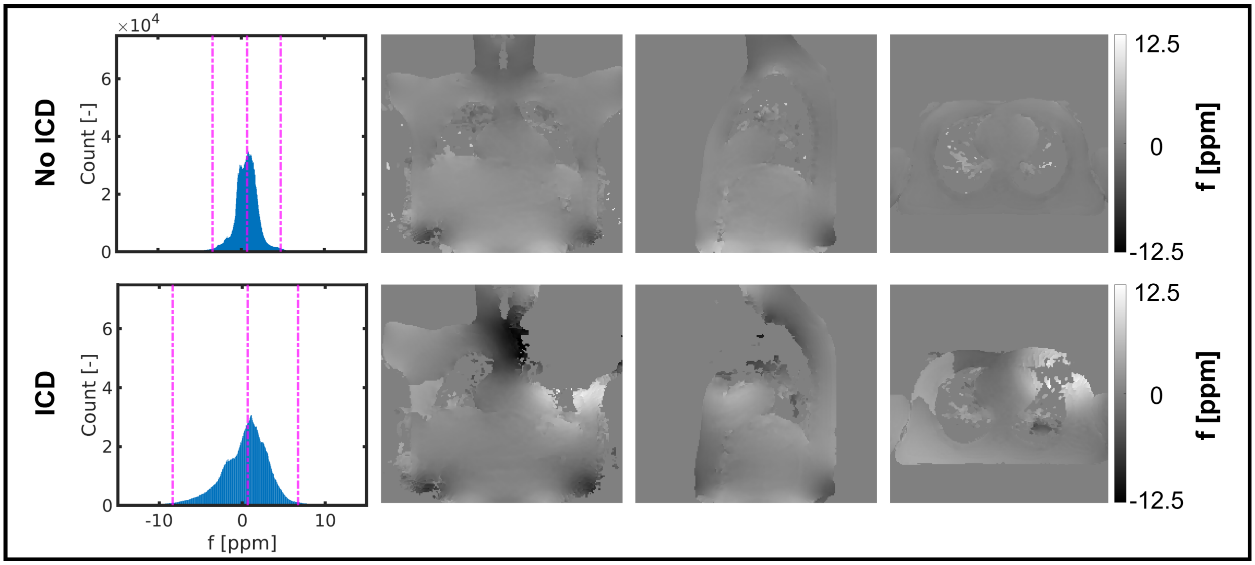

Figure S-7: A single slice in each orthogonal imaging plane of the unwrapped B_0-map in a single healthy volunteer (V5) without an ICD (top row) and with an ICD (bottom row) with the corresponding histogram of measured frequency offsets within the body contour.

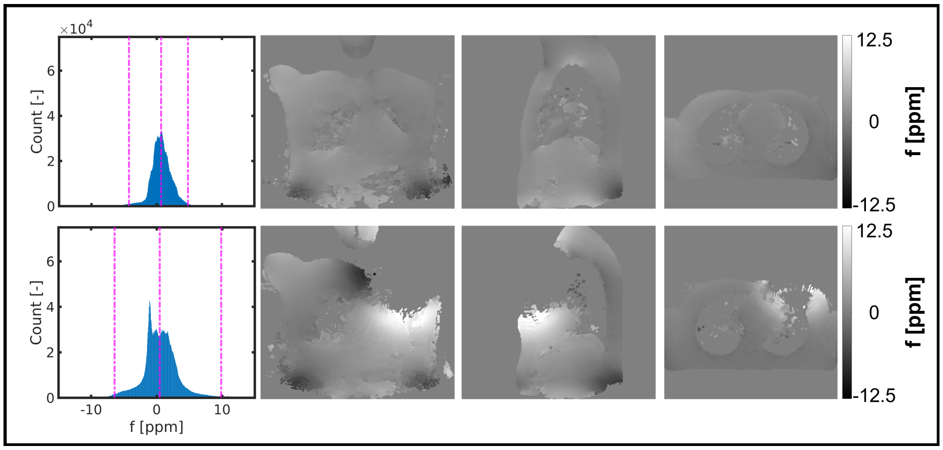

Figure S-8: A single slice in each orthogonal imaging plane of the unwrapped B_0-map in a single healthy volunteer (V1) without an ICD (top row) and with an ICD (bottom row) with the corresponding histogram of measured frequency offsets within the whole acquisition volume.

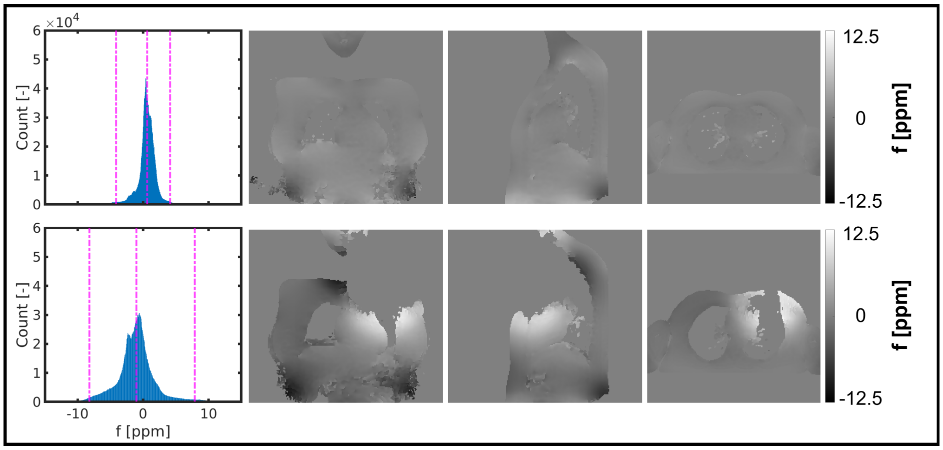

Figure S-9: A single slice in each orthogonal imaging plane of the unwrapped B_0-map in a single healthy volunteer (V2) without an ICD (top row) and with an ICD (bottom row) with the corresponding histogram of measured frequency offsets within the whole acquisition volume.

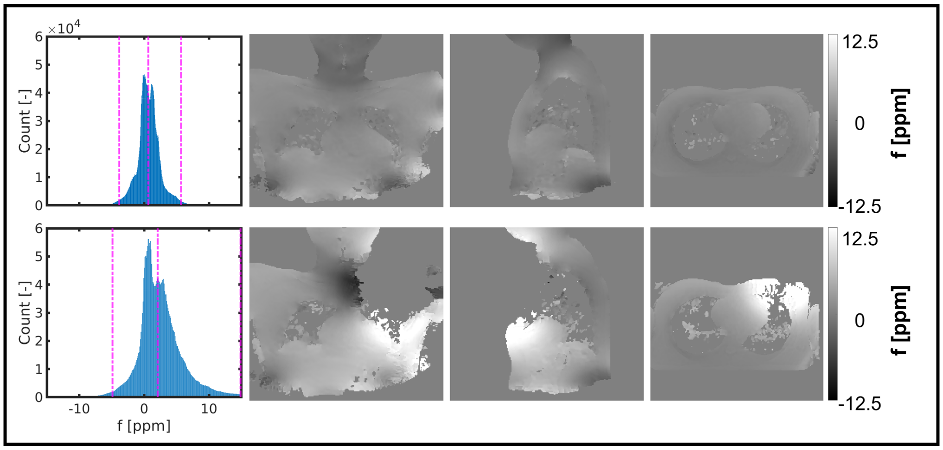

Figure S-10: A single slice in each orthogonal imaging plane of the unwrapped B_0-map in a single healthy volunteer (V3) without an ICD (top row) and with an ICD (bottom row) with the corresponding histogram of measured frequency offsets within the whole acquisition volume.

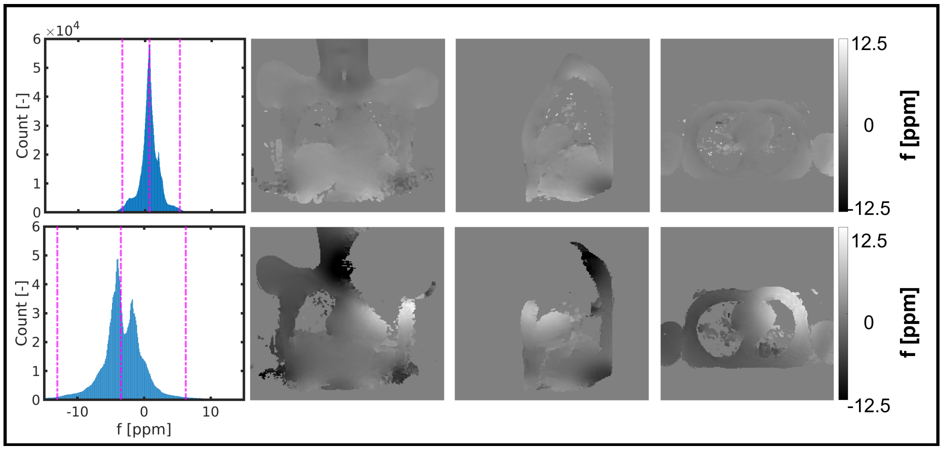

Figure S-11: A single slice in each orthogonal imaging plane of the unwrapped B_0-map in a single healthy volunteer (V4) without an ICD (top row) and with an ICD (bottom row) with the corresponding histogram of measured frequency offsets within the whole acquisition volume.
